# Akebia Saponin D Regulates the Metabolome and Intestinal Microbiota in High Fat Diet-Induced Hyperlipidemic Rats

**DOI:** 10.3390/molecules24071268

**Published:** 2019-04-01

**Authors:** Peipei Zhou, Xiaolin Yang, Zhonglin Yang, Wenzhe Huang, Junping Kou, Fei Li

**Affiliations:** 1 State Key Laboratory of Natural Medicines, China Pharmaceutical University, Nanjing 210009, China; zppcpu@163.com (P.Z.); yzl1950@126.com (Z.Y.); 2 Key Laboratory of Pharmaceutical and Biological Marine Resources Research and Development of Jiangsu Province, Nanjing University of Chinese Medicine, Nanjing 210009, China; xiaolinysn@126.com; 3 Nanjing Research Institute, Jiangsu Kangyuan Pharmaceutical Co., LTD, Nanjing 211100, China; njhwzh@hotmail.com; 4 Jiangsu Key laboratory of TCM Evaluation and Translational Research, Department of Complex TCM Prescriptions, China Pharmaceutical University, Nanjing 211198, China

**Keywords:** akebia saponin D, metabolomics, intestinal microbiota, hyperlipidemia, UPLC-Q/TOF-MS, 16s rRNA

## Abstract

Hyperlipidemia is a major component of metabolic syndrome, and regarded as one of the main risk factors causing metabolic diseases. We have developed a therapeutic drug, akebia saponin D (ASD), and determined its anti-hyperlipidemia activity and the potential mechanism(s) of action by analyzing the metabolome and intestinal microbiota. Male Sprague-Dawley rats were fed a high fat diet to induce hyperlipidemia, and then given ASD orally for 8 weeks. Lipid levels in serum were determined biochemically. Metabolites in serum, urine and feces were analyzed by UPLC-Q/TOF-MS, and the structure of the intestinal microbiota was determined by 16S rRNA sequencing. The ASD treatment significantly decreased the levels of TC, TG and LDL-c and increased the serum level of HDL-c. Metabolomics analysis indicated that the ASD treatment mainly impacted seven differential metabolites in the serum, sixteen differential metabolites in the urine and four differential metabolites in feces compared to the model group. The ASD treatment significantly changed eight bacteria at the genus level compared to the model group. In conclusion, ASD treatment can significantly alleviate HFD-induced hyperlipidemia and the hypolipidemic effect of ASD treatment is certainly associated with a systematic change in the metabolism, as well as dynamic changes in the structure of the intestinal microbiota.

## 1. Introduction

**Figure 1:**
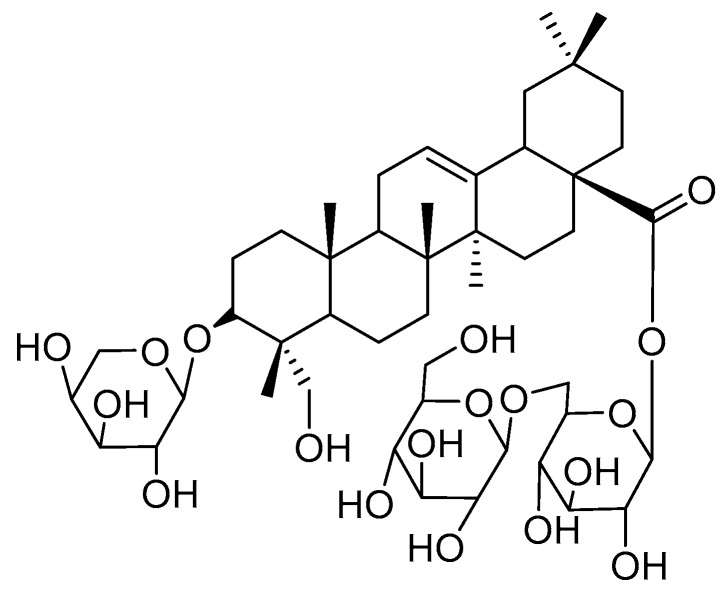
Chemical structure of ASD.

Hyperlipidemia is a major public health problem and refers to several serum (or plasma) lipid component abnormalities, including increased levels of triglyceride (TG) and/or total cholesterol (TC), low-density lipoprotein cholesterol (LDL-c), and decreased high-density lipoprotein cholesterol (HDL-c) [1]. Hyperlipidemia is regarded as one of the main risk factors that cause metabolic diseases including atherosclerosis, hypertension, type 2 diabetes and coronary heart disease [2,3]. Commonly used hypolipidemic drugs included statins, fibrates, nicotinic acid, cholesterol absorption inhibitors and bile acid sequestrants [4]. Many of these medications have significant adverse effects, such as statin myopathy, liver injury, sleep disturbance and rhabdomyolysis [5,6]. Hence, the development of safe and effective anti-hyperlipidemia drugs is particularly significant and urgent. Akebia saponin D (ASD, Figure 1), a typical bioactive triterpenoid saponin isolated from the rhizome of *Dipsacus asper* Wall, has been reported to have multiple therapeutic properties, including cardioprotective [7,8,9], anti-Alzheimer’s disease [10,11,12,13,14]. anti-osteoporosis [15,16,17], and non-alcoholic fatty liver disease (NAFLD) treatment effects [18,19,20].

In this study, we firstly examined the activity of ASD in the treatment of hyperlipidemia using a high fat diet-induced rat model, then, determined the metabolome using Ultra Performance Liquid Chromatography quadrupole time-of-flight mass spectrometry (UPLC-Q/TOF-MS) [21,22,23,24], and determined the structure of the intestinal microbiota by 16S rRNA sequencing [25,26,27,28] to discuss the underlying mechanism(s).

## 2. Results

### 2.1. The Effect of ASD Treatment on Lipid Level in HFD-Induced Hyperlipidemia Rats

**Figure 2:**
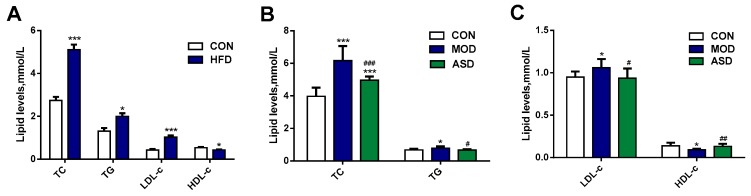
Effect of high fat diet and ASD treatment on lipid levels of TC, TG, LDL-c and HDL-c. (**A**) the lipid levels after feeding high fat diet for 4 weeks; (**B**,**C**) the lipid levels after ASD treatment for 8 weeks. The data was presented as the mean ± SD, * *p* < 0.05, *** *p* < 0.001 vs. the chow diet group; ^#^ *p* < 0.05, ^##^ *p* < 0.01, ^###^ *p* < 0.001 vs. the model group. (CON = the chow diet group, n = 8; HFD = high fat diet group, n = 16; MOD = the model group, n = 8; ASD = ASD treated group, n = 8).

Male SD rats developed hyperlipidemia after four weeks of HFD feeding. As shown in Figure 2, the serum TC, TG, and LDL-c levels of the HFD group were increased, whereas HDL-c levels decreased significantly as compared to the chow diet group (Figure 2A). After 8-weeks of ASD treatment, the serum TC, TG, and LDL-c levels were decreased, whereas HDL-c levels were significantly increased as compared to the model group (Figure 2B,C). These data indicated that the HFD-induced hyperlipidemia model was successfully established and the ASD treatment effectively reversed this condition.

### 2.2. Metabolomics Analysis of ASD Treatment in HFD-Induced Hyperlipidemia Rats

#### 2.2.1. The Effect of ASD Treatment on Serum Metabolites

**Figure 3:**
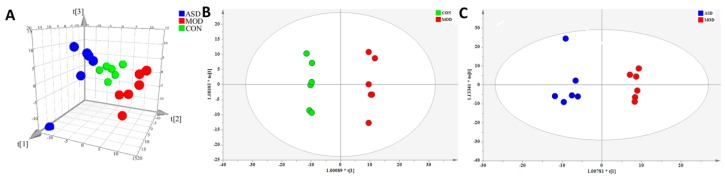
PLS-DA (**A**) and OPLS-DA (**B**,**C**) score plot of serum samples discrimination of the metabolome between three groups, the chow diet group vs. model group vs. ASD treated group (**A**), the model group vs. chow diet group (**B**), the ASD treated group vs. model group (**C**). (CON = the chow diet group, n = 6; MOD = high-fat diet model group, n = 6; ASD = ASD treated group, n = 6).

Representative total ion chromatograms (TIC) of serum samples are shown in Figure S1 (Supplementary Material File 1). A total of 470 metabolite ions in both positive and negative ion modes were extracted from all serum samples, and after normalization, 467 ions remained. Using PLS-DA and OPLS-DA (Figure 3), obvious differences in serum metabolic phenotypes between the three groups was observable. The R2Y and Q2 of PLS-DA analyses scores of the three groups were 0.98 and 0.885 (Figure 3A), respectively. The R2Y and Q2 of OPLS-DA analyses scores were 0.995 and 0.912 (Figure 3B; the model group vs. chow diet group), and 0.97 and 0.787 (Figure 3C; the ASD treated group vs. model group), respectively. These data indicated the validity of the current models. Based on VIP > 1 and *p* value < 0.05, 22 common metabolites between the model group and chow diet group samples, and seven metabolites between the ASD treatment group and model group samples were found, as shown in Table 1. The results showed that ASD treatment reversed the variation trend of almost total 31 differential metabolites which were altered by a high fat diet.

**Figure 4:**
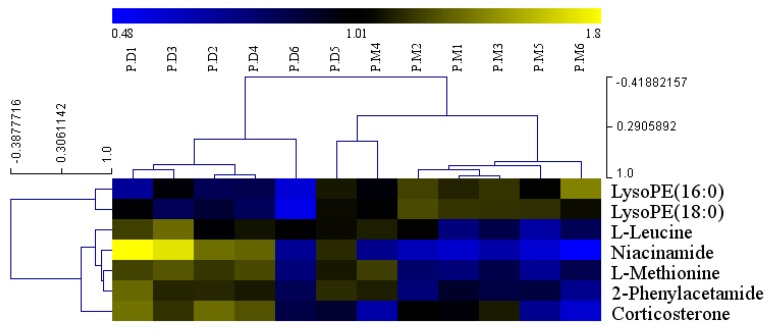
Heatmap visualization of the differential metabolites identified of the serum samples from ASD treated group and model group. PM1, PM2, PM3, PM4, PM5, PM6 are from the model group. PD1, PD2, PD3, PD4, PD5, PD6 are from the ASD treated group.

We next determined the effect of ASD treatment on serum metabolites in hyperlipidemic rats by comparison with the model group. The mean normalized quantities of the differential metabolites identified between the ASD treatment and model group in serum samples are displayed as a heatmap in Figure 4. As shown in Figure S2 (Supplementary Material File 1), among the increased metabolites, l-methionine is involved in cysteine and methionine metabolism and aminoacyl-tRNA biosynthesis, niacinamide is involved in nicotinate and nicotinamide metabolism, l-leucine is involved in the biosynthesis of valine, leucine and isoleucine and degradation of valine, leucine and isoleucine, aminoacyl-tRNA biosynthesis, 2-phenylacetamide is involved in phenylalanine metabolism, corticosterone is involved in steroid hormone biosynthesis. The decreased metabolites, such as LysoPE (16:0) and LysoPE (18:0) are involved in glycerophospholipid metabolism.

#### 2.2.2. The Effect of ASD Treatment on Urine Metabolites

**Figure 5:**
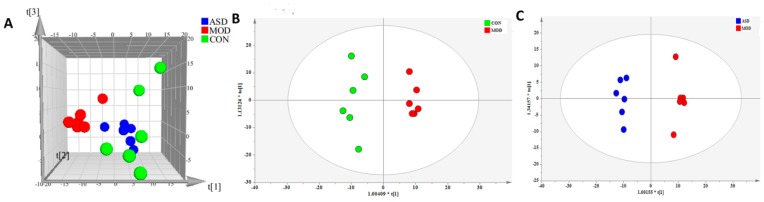
PLS-DA (**A**) and OPLS-DA (**B**,**C**) score plot of urine samples discrimination of the metabolome between three groups, the chow diet group vs. model group vs. ASD treated group (**A**), the model group vs. chow diet group (**B**), the ASD treated group vs. model group (**C**). (CON = the chow diet group, n = 6; MOD = high-fat diet model group, n = 6; ASD = ASD treated group, n = 6).

Representative TICs of urine samples are shown in Figure S3 (Supplementary Material File 1). A total of 531 metabolite ions in both positive and negative ion modes were extracted, and after normalization, 515 ions remained. Using PLS-DA and OPLS-DA (Figure 5), the three groups were clearly classified. The R2Y and Q2 of PLS-DA analyses scores of the three groups were 0.89 and 0.707 (Figure 5A), respectively. The R2Y and Q2 of OPLS-DA analyses scores were 0.969 and 0.798 (Figure 5B) (the model group vs. chow diet group), and 0.986 and 0.774 (Figure 5C) (the ASD treated group vs. model group), respectively. These data indicated the validity of the current models. Based on VIP > 1 and *p* value < 0.05, seven differential metabolites between the model group and chow diet group samples, and 16 differential metabolites between the ASD treatment group and model group samples were found, as shown in Table 2, and we found that ASD treatment can reverse the variation trend of almost total 19 differential metabolites which were altered by the high fat diet.

**Figure 6:**
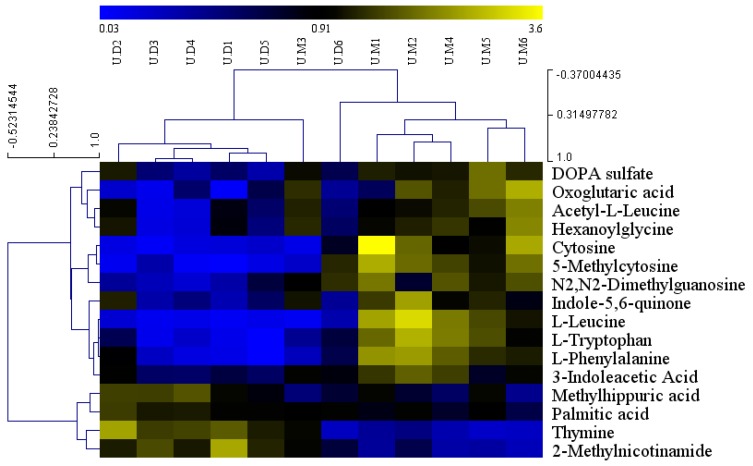
Heatmap visualization of the differential metabolites identified of the urine samples from ASD treated group and model group. UM1, UM2, UM3, UM4, UM5, UM6 are from the model group. UD1, UD2, UD3, UD4, UD5, UD6 are from the ASD treatment group.

We next determined the effect of ASD treatment on urine metabolites by comparison with the model group. The mean normalized quantities of the differential metabolites identified between the ASD treated and model group in urine samples are displayed as a heatmap in Figure 6. The ASD treatment was resulted in increases in four metabolites (including 2-methylnicotinamide, thymine, and palmitic acid) and decreases of 15 metabolites (including oxoglutaric acid, l-leucine, l-phenylalanine, l-tryptophan, DOPA sulfate, indole-5,6-quinonebin, hexanoylglycine, and 3-indole-acetic acid) in the urine samples (Table 2). Notably, the metabolites in urine samples was mainly related to amino acid metabolism (Figure S4, Supplementary Material File 1), phenylalanine, tyrosine and tryptophan biosynthesis, phenylalanine metabolism, tryptophan metabolism, valine, leucine and isoleucine biosynthesis, alanine, aspartate and glutamate metabolism.

#### 2.2.3. The Effect of ASD Treatment on Feces Metabolites

**Figure 7:**
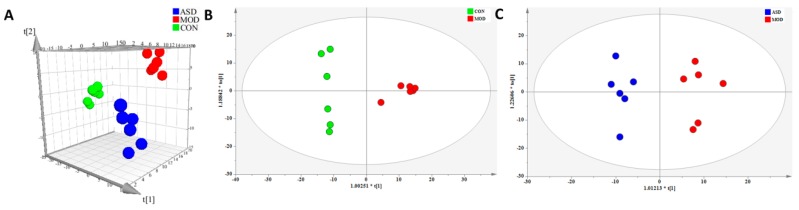
PLS-DA (**A**) and OPLS-DA (**B**,**C**) score plot of feces samples discrimination of the metabolome between three groups, the chow diet group vs. model group vs. ASD treated group (**A**), the model group vs. chow diet group (**B**), the ASD treated group vs. model group (**C**). (CON = the chow diet group, n = 6; MOD = high-fat diet model group, n = 6; ASD = ASD treated group, n = 6).

Representative TIC of feces samples are shown in Figure S5 (Supplementary Material File 1). A total of 640 metabolite ions in both positive and negative ion modes were extracted, and after normalization, 445 ions remained. Using PLS-DA and OPLS-DA (Figure 7), obvious differences in feces metabolic phenotypes existed between the three groups. The R2Y and Q2 of PLS-DA analyses scores of the three groups were 0.855 and 0.625 (Figure 7A), respectively. The R2Y and Q2 of OPLS-DA analyses scores were 0.954 and 0.849 (Figure 7B; the model group vs. chow diet group), and 0.94 and 0.729 (Figure 7C; the ASD treated group vs. model group), respectively. These data indicated the validity of the current models. Based on VIP > 1 and *p* value < 0.05, three differential metabolites between the model group and chow diet group rats, and four differential metabolites between the ASD treatment group and model group rats were identified, as shown in Table 3.

**Figure 8:**
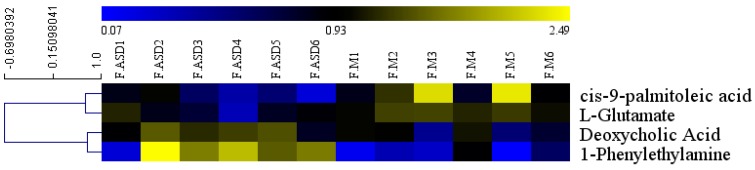
Heatmap visualization of the differential metabolites identified of the feces samples from ASD treated group and model group. FM1, FM2, FM3, FM4, FM5, FM6 are from the model group. FASD1, FASD2, FASD3, FASD4, FASD5, FASD6 are from the ASD treatment group.

The mean normalized quantities of the differential metabolites identified between the ASD treatment and model group in feces samples are displayed as a heatmap in Figure 8. The ASD treatment resulted in two increased metabolites (1-phenylethylamine and deoxycholic acid) and two decreased metabolites (L-glutamate, and *cis*-9-palmitoleic acid) in the feces samples (Table 3).

Among the metabolites, the related major metabolic patterns are d-glutamine and d-glutamate metabolism, alanine, aspartate and glutamate metabolism, glutathione metabolism, arginine and proline metabolism (Figure S6, Supplementary File 1).

### 2.3. The Effect of ASD Treatment on the Structure of Intestinal Microbiota in Feces

**Figure 9:**
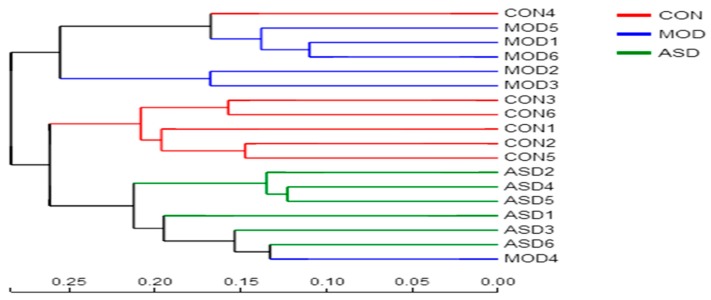
Hierarchical clustering at the genus level of the microbiota composition among the three groups. (CON = the chow diet group, n = 6; MOD = high-fat diet model group, n = 6; ASD = ASD treated group, n = 6).

**Figure 10:**
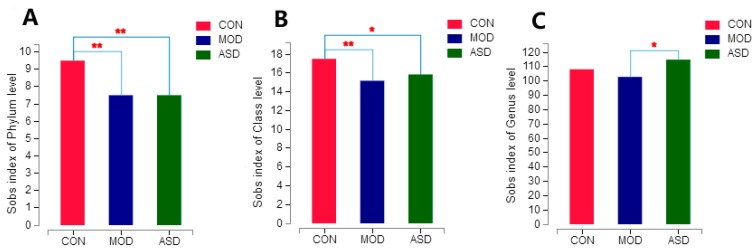
Comparison of the α-diversity at phylum (**A**), class (**B**) and genus (**C**) levels among the three groups based on the SOBS index by using Student’s *t* test, * *p* < 0.05, ** *p* < 0.01. (CON = the chow diet group, n = 6; MOD = high-fat diet model group, n = 6; ASD = ASD treated group, n = 6).

To determine the effect of ASD treatment on the intestinal microbiota composition, we conducted Illumina MiSeq 16S rRNA amplicon sequencing, which produced 836,659 valid sequences from 18 samples. According to the minimum sample sequence quantity, the extraction processing was carried out all the samples to 22,891 sequences per sample for the next analysis. As depicted in Figure S7 (Supplementary Material, File 1) the rarefaction curves demonstrated that there were sufficient sequencing sampling reads to perform a meaningful analysis. The Bray-Curtis PCoA method was applied to visually assess the similarities or dissimilarities in the gut microbiota among all of the samples. As shown in Figure S8 (Supplementary Material, File 1) the microbiota composition of the three groups were clearly classified. Cluster analysis revealed that the samples in the chow diet group, model group and ASD treated group formed separate clusters from each other on the genus level (Figure 9). Alpha-diversity analysis based on the SOBS index by using Student’s *t* test demonstrated that the model group and ASD treatment group showed significantly lower community richness compared to that of the chow diet group on a phylum (Figure 10A) and class (Figure 10B) level. Meanwhile, the ASD treatment group was showed a significantly higher community richness compared to that of the model group on a genus level (Figure 10C). The results above indicate that the decreased diversity of the intestinal microbiota was linked with the high fat diet and the treatment of ASD could partly but significantly reverse the changes.

**Figure 11:**
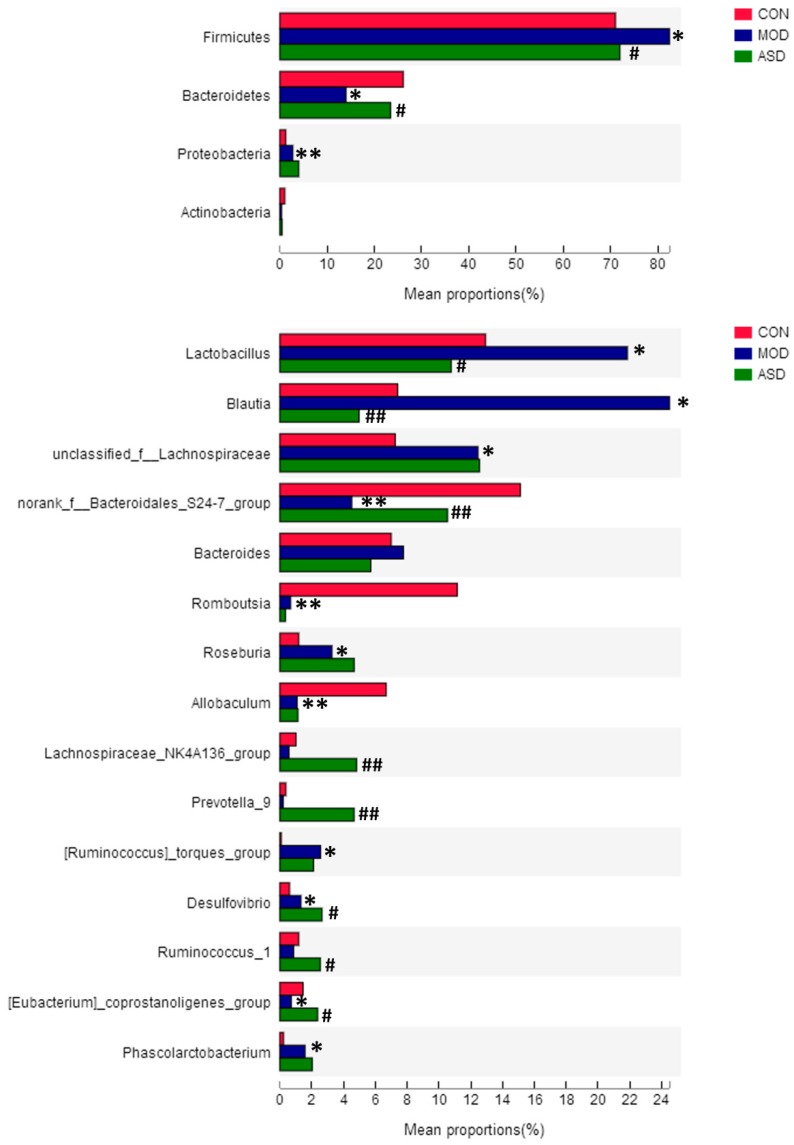
The variation analysis of microbiota composition at phylum (**A**) and genus (**B**) level among the three groups. (* *p* < 0.05, ** *p* < 0.01 vs. the chow diet group; ^#^ *p* < 0.05, ^##^ *p* < 0.01 vs. the model group. CON = the chow diet group, n = 6; MOD = high-fat diet model group, n = 6; ASD = ASD treated group, n = 6).

Then, we performed the microbial differences analysis and correlation analysis using I-Sanger. The community heatmap of key OTUs based on genus-level changes among the three groups is shown in Figure S9 (Supplementary Material, File 1). As shown in Figure 11, at the phylum level (Figure 11A), the majority of the OTUs belonged to Firmicutes and Bacteroidetes, followed by Proteobacteria and Actinobacteria. The model group showed significantly increased relative abundance of Firmicutes and Proteobacteria, and decreased Bacteroidetes and Actinobacteria compared with chow diet group. Meanwhile, the abundances of the majority phyla (Firmicutes and Bacteroidetes) were significantly altered by ASD treatment compared with the model group. At the genus level (Figure 11B), the model group showed significantly increased relative abundance of Lactobacillus, Blautia, unclassified Lachnospiraceae, Roseburia, *Ruminococcus_torques*_group, Desulfovibrio, and Phascolarctobacterium, decreased norank_f_Bacteroidales_S24-7_group, Romboutsia, Allobaculum, and *Eubacterium_coprostanoligenes*_group compared with chow diet group based on the top fifteen genus. The ASD-treated group showed significantly increased relative abundance of norank_f_Bacteroidales_S24-7_group, Lachnospiraceae_NK4A136_group, Prevotella_9, Desulfovibrio, Ruminococcus_1 and *Eubacterium_coprostanoligenes*_group, and decreased Lactobacillus and Blautia compared with the model group. The above results show that the treatment with ASD resulted in partial changes in the intestinal microbiota, and the samples were more similar to those of the chow diet group compared to the model group based on the major microflora present.

### 2.4. The Correlation between Fecal Metabolites and Microbiota

**Figure 12:**
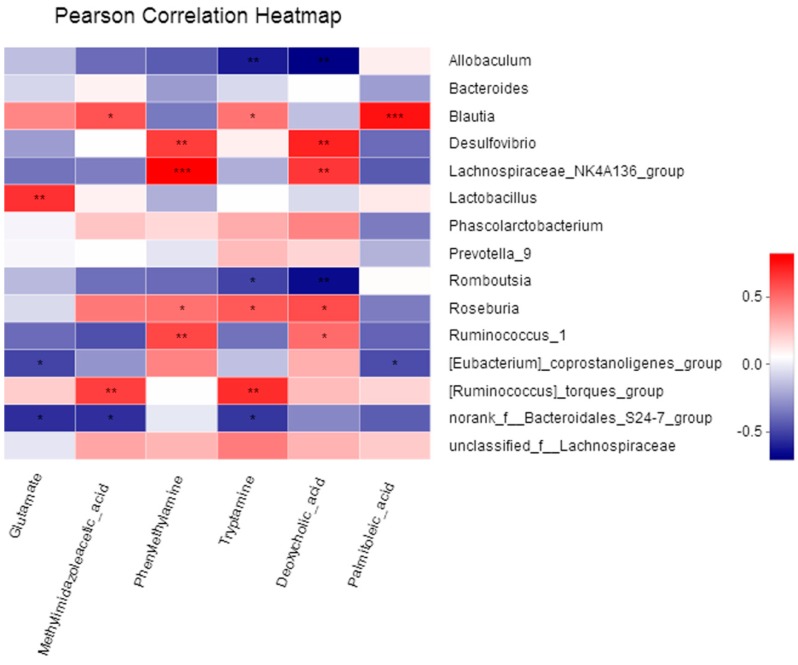
Pearson correlation between fecal metabolites and microbiota (at the genus level) affected by ASD treatment. Red color indicating positive correlations whereas blue denoting negative correlations (* *p* < 0.05, ** *p* < 0.01, *** *p* < 0.001).

Correlation between fecal metabolites and microbiota in the rats from three groups was also investigated in the present study. Pearson correlation between the selected metabolites and the fecal microbiota relative abundance at the genus level was analyzed. Interestingly, a clear correlation with the fecal metabolites was found for the disturbed fecal microbiota at a genus level. As shown in Figure 12, l-glutamate showed a positive correlation with Lactobacillus and a negative correlation with the norank_f_Bacteroidales_S24-7_group and *Eubacterium_coprostanoligenes*_group. 1,4-Methyl-imidazoleacetic acid showed a positive correlation with the *Ruminococcus_torques*_group, and Blautia and a negative correlation with norank_f_Bacteroidales_S24-7_group. 1-Phenylethylamine showed a positive correlation with Ruminococcus_1, Roseburia, Lachnospiraceae_NK4A136_group and Desulfovibrio. Tryptamine showed a positive correlation with the *Ruminococcus_torques*_group, Roseburia and Blautia, and showed negative correlation with norank_f_Bacteroidales_S24-7_group, Romboutsia and Allobaculum. Deoxycholic acid showed a positive correlation with Ruminococcus_1, Roseburia, Lachnospiraceae_NK4A136_group and Desulfovibrio, and showed a negative correlation with Romboutsia and Allobaculum. *cis*-9-Palmitoleic acid showed a positive correlation with Blautia and a positive correlation with the *Eubacterium_coprostanoligenes*_group.

## 3. Discussion

Hyperlipidemia is one of the components of metabolic syndrome, and the pathophysiology is very complex and has been only partially elucidated. As in previous unpublished studies and our study reported in this article, HFD feeding significantly promoted the development of hyperlipidemia. Meanwhile, ASD treatment significantly alleviated the HFD-induced hyper-lipidemia. However, there seems to be a paradox between the high efficacy and the low oral bioavailability of ASD. ASD is poorly absorbed when administrated orally and the oral bioavailability of ASD in rats was only 0.13% [29,30,31] due to metabolism in the gastrointestinal tract, excretion from the bile, feces and decomposition in the liver. This phenomenon prompted us to hypothesize that ASD exert its hypolipidemic effects primarily by two different mechanisms. One is the maintenance of dynamic equilibrium of metabolites by interfering with key metabolic pathways. The other is the modulation of intestinal microbiota to further attenuate the metabolic syndrome. Thus, in the current study, we have tried to determine the underlying mechanisms of the hypolipidemic effects of ASD by researching the metabolomics and intestinal microbiota.

Metabolomics is a new science that aims to quantitatively describe the dynamic changes of many metabolites in organisms. Some specific metabolites can reflect the metabolic characteristics of the individual and the disease [32]. By applying integrated metabolomics in serum, urine and feces, we identified a number of metabolites in hyperlipidemic rats treated with ASD. In serum samples, the differential metabolites were mainly (impact-value threshold above 0.10) [33] involved in valine, leucine and isoleucine biosynthesis (l-leucine) and nicotinate and nicotinamide metabolism (niacinamide). In urine samples, the differential metabolites were mainly involved in phenylalanine, tyrosine and tryptophan biosynthesis (l-phenylalanine), phenylalanine metabolism (l-phenylalanine), valine, leucine and isoleucine biosynthesis (l-leucine) and tryptophan metabolism (l-tryptophan). In feces samples, the differential metabolites were mainly involved in d-glutamine and d-glutamate metabolism and alanine, aspartate and glutamate metabolism (l-glutamate).

The most obvious finding of our analysis was that the major metabolites were related to amino acid metabolism, suggesting that amino acid metabolism is one of the focuses of hyperlipidemia. It has been reported in the literature [34] that abnormalities in amino acid metabolism are associated with cardiovascular diseases and are one of the basic metabolic pathways in the body. Ketogenic amino acids, glycogen and ketogenic amino acids can generate acetyl-CoA in the metabolic process. Glycogen amino acids can be directly or indirectly generated into pyruvate, which can not only be converted into glycerol, but also can be generated into acetyl-CoA through oxidative decarboxylation. Acetyl-CoA can be regenerated into fatty acids by the fatty acid synthesis pathway.

Leucine is a kind of branched-chain amino acid, which animals cannot synthesize by themselves and ingest with their diet. Leucine plays an important role in promoting protein synthesis, glucose metabolism and oxidation, and regulating immune function and fat metabolism. High levels of leucine contribute to decreased the fatty acid synthase (FAS) gene expression and the activity of FAS [35,36]. A recent study has reported that an increase of leucine in NCI-H716 human colon cancer cells can result in downregulated expression of genes related to the transport and combination of intestinal fatty acids, such as Niemann-Pick C-1-like-l protein (NPC1Ll), acetyl-co-enzyme A carboxylase (ACC), FAS, sterol regulatory element-binding protein-2 (SREBP-2), 3-hydroxy-3-methylglutaryl- CoA reductase (HMGCR) and fatty acid transport protein 4 (FATP4), further decreasing the synthesis of fat [37]. ASD treatment significantly increased the leucine levels in serum and decreased the level in urine, which might be contribute to its hypolipidemic effects.

Phenylalanine is a kind of ketogenic amino acid, involved in phenylalanine, tyrosine and tryptophan biosynthesis, which generally is first converted into tyrosine in organisms. Phenylalanine and tyrosine are the precursors of epinephrine and other catecholamines, and consumption increases when epinephrine is needed for lipid metabolism [38]. Tyrosine is transformed into 4-hydroxy-phenylpyruvate (HPPA), and further oxidized into homogentisic acid by 4-hydroxyphenylpyruvate dioxygenase (HPPD), and fumaric acid and acetoacetic acid are produced after a series of metabolic reactions, and then participate in the tricarboxylic acid cycle (TAC cycle). Acetylcoenzyme A involved in the TAC cycle can synthesize fatty acids. The results showed that ASD relief in lipid metabolism disorders may be related to its regulation of phenylalanine, tyrosine and tryptophan metabolism, increased amounts of tyrosine production, and reduced phenylalanine.

Tryptophan is also a kind of ketogenic amino involved in tryptophan metabolism and has the function of regulating fat metabolism [39]. Tryptophan is the precursor of the biosynthesis of the neurotransmitter serotonin (5-hydroxytryptamine, 5-HT) which is a biochemical messenger and regulator. Tryptophan is mainly metabolized by the 5-HT and the kynurenine (KYN) pathways. 4,6-Dihydroxyquinoline (quinoline-4,6-diol) and formylanthranilate are the products of the two pathways, respectively. Under normal physiological conditions, tryptophan can not only serve as an amino acid substrate to participate in the various protein synthesis processes in body tissue cells, but also regulate the protein synthesis process [40]. Tryptophan is involved in the TCA circle by being converted into pyruvate, which is converted to glycerol, and the glycerol and fatty acid can synthesis fatd. ASD treatment decreased l-tryptophan in urine, which may contribute to its hypolipidemic effects.

Glutamate is a kind of the gluconeogenic amino acid and the precursor of glutathione. Glutamate is the first line of defense against free radicals in the liver, and it is an essential amino acid during the pathogenesis of metabolic diseases [41]. Although the in vivo increase of hepatic glutamate in our study was not significant, the glutamate level in feces markedly decreased, indicating a reduced degradation and secretion of glutamate. This finding demonstrated that the hypolipidemic effects of ASD administration might be related to d-glutamine and d-glutamate metabolism.

Niacinamide, also called vitamin B3 or vitamin PP, is regarded as one of the important components of coenzyme I (nicotinamide adenine dinucleotide, NAD) and coenzyme II (nicotinamide adenine dinucleotide phosphate, NADP). These two coenzymes play important roles in biological oxidation and metabolism. Niacinamide is a metabolite of niacin produced by the amidation pathway, that can inhibit the release of free fatty acids in fat tissue and increase the activity of lipoprotein enzymes and decrease the synthesis rate of LDL-c, as well as reduced the TC, TG, and increase HDL-c [42]. During the regulation of lipid metabolism disorders, the content of niacinamide increased to some extent, indicating that the ASD can relieve the dyslipidemia symptoms by promoting the metabolism of niacin and niacinamide and increasing the content of niacinamide.

A healthy intestinal microbiota is greatly important for the health of the host [43]. Mounting evidence shows that the alteration of the intestinal microbiota composition contributes to the development of metabolic disorders [44,45], including lipid and cholesterol metabolism problems. In the present study, we found differences in the intestinal microbiota between HFD-fed and normal chow-fed rats, and this finding supported the conclusion that intestinal microbial communities were associated with hyperlipidemia progression. Furthermore, we demonstrated that ASD alleviated the HFD-induced hypolipidemic and showed that the intestinal microbiota composition in the ASD group differed from that in the model group. Our results indicated that modulating the intestinal microbiota composition could be a future pharmacological approach for protection against hypolipidemia.

Firmicutes, Bacteroidetes, Proteobacteria, and Actinobacteria were the dominant microbial divisions in the samples. Our observation was that the treatment of ASD significantly reversed the changes of Bacteroidetes and Firmicutes induced by HFD feeding, ultimately being almost similar to the chow diet group. It has been reported that the abundances of Firmicutes and Bacteroidetes in the intestinal microbiota were associated to lipid metabolism and energy homeostasis of the host [46]. At a genus level, ASD treatment modified the levels of some specific bacteria that were altered in the model group rats. Firstly, ASD treatment reversed the relative abundance of top two genus (Lactobacillus and Blautia) induced by high fat diet. Secondly, the ASD-treated group showed a markedly significant increased in the relative abundance of norank_f_Bacteroidales_S24-7_group, Lachnospiraceae_NK4A136_group and Ruminococcus_1 which are short-chain fatty acid (SCFA)-producing bacteria [47,48,49], that not only provide energy for the intestinal mucosa cells and promote the metabolism and growth of cells in the colon but also lower the environmental pH of the colon, reduce the growth of harmful bacteria and prevent intestinal dysfunction. According to a recent study, SCFA might contribute to obesity by increasing the host’s capacity for energy harvesting from foods [50]. Prevotella are also SCFA-producing bacteria, which are able to produce propionic acid (as main metabolite). Recent studies have shown that Prevotella was associated with diets rich in carbohydrates and simple sugars [51], and increased levels of Prevotella and Prevotella/Bacteroides ratio may be advantageous with respect to postprandial glucose homeostasis [47]. Taken together, the beneficial effects of SCFAs, namely improving gut barrier functions, ameliorating systemic inflammation, or creating a non-permissive environment for pathogens [52], might mediate the pharmacological effects of ASD against hyperlipidemia. Desulfovibrio is a sulfate-reducing bacteria (SRB) that utilizes lactic acid, pyruvate, ethanol, and fatty acids to reduce sulfate into hydrogen sulfide (H_2_S) [53]. H_2_S may have a toxic effect on intestinal epithelial cells, hindering the butyrate oxidation pathway in colon cells and causing apoptosis and chronic inflammation [54]. A higher abundance of Desulfovibrio was found in offspring of obese mothers, which might be a gut microbiota-mediated mechanism for diabetogenesis by maternal obesity [55], and further impacts lipid metabolism. In this study, a surprised finding was that the administration of ASD increased the abundance of Desulfovibrio in hyperlipidemia mice, which indicated that ASD hypolipidemic effects might be related to the global change of the intestinal microbiota, not only to Desulfovibrio. Meanwhile, HFD diet decreased the richness of *Eubacterium_coprostanoligenes* in this study, and this tendency was reversed by ASD treatment. The result above suggested that ASD is expected to exert hypolipidemic effects through maintaining and modulating the intestinal microbiota.

Moreover, correlations were observable between the fecal metabolites and microbiota. A great number of studies have confirmed the correlations between gut microbiota and metabonomics in HFD-treated animals [25,56]. Our results showed that there was a possible link between the altered microbiota and metabolites in ASD-treated rats. ASD made a significant difference the in fecal metabolites, and these changed metabolites might affect genera abundance. However, the sophisticated mechanism relating endogenous metabolites and microbes affected by ASD treatment has not been clearly elucidated. In future, more studies are needed to elucidate the interactions between ASD and specific bacterial genera.

## 4. Materials and Methods

### 4.1. Chemicals and Reagents

ASD (93.4%, HPLC purity) was a pilot product produced by our laboratory. ASD purity was determined based on the standard substance purchased from the National Institutes for Food and Drug Control (Beijing, China). Pure water was obtained from a GenPure Pro UV/UF Laboratory Water System (Thermo, Waltham, MA, USA). Methanol, acetonitrile, formic acid and ammonium acetate for HPLC grade were purchased from Merck Chemicals (Darmstadt, Germany). Sodium carboxymethylcellulose (CMC–Na) was purchased from Sinopharm Chemical Reagent Co. Ltd. (Shanghai, China). TG, TC, LDL-c and HDL-c kit were purchased from Nanjing Jiancheng Biotechnology Co. Ltd. (Nanjing, China). OMEGA-soil DNA Extration Kit (Omega Bio-Tek, Norcross, GA, USA), FastPfu Polymerase (TransGen, Beijing, China) Axygen Biosciences Axy Prep DNA Gel Extraction Kit (Axygen, Corning, NY, USA), Tru Seq™ DNA Sample Prep Kit Illumina MiSeq platform (Illumina, San Diego, CA, USA).

### 4.2. Animal Treatment

Male Sprague-Dawley (SD) rats (8-week old, 180–220 g), purchased from Shanghai Jiesijie Laboratory Animal Co., Ltd. (Shanghai, China, certification No. SCXK (Hu) 2018-0004], were maintained under standardized conditions (22 ± 2°C, 60 ± 5% humidity, 12 h day/night cycle). After 1-week acclimatization, rats were randomly divided into a chow diet group (n = 8) and a high fat diet (HFD; 10% lard, 10% yolk powder, 2% cholesterol, 1% bile salt and 77% standard chow) group (n = 16). Blood samples were collected from the orbital venous plexus after 4 weeks of feeding, and TC, TG, LDL-c, HDL-c levels in serum were quantified. The HFD group rats were further divided into a model group (continued to supply HFD, n = 8) and ASD-treated group (with HFD and ASD supplementation, n = 8). ASD was suspended with 0.5% CMC-Na solution, and then administered by oral gavage for the next 8 weeks, administration dosage was 90 mg/kg. The chow diet group and model group rats were received an equal volume 0.5% CMC-Na solution. To collect urine and feces samples, six rats in each experimental group were housed in metabolic cages, and the samples accumulated in the metabolic cages were transferred into sterile tubes, snap-frozen in liquid nitrogen, and then stored at −80 °C. At the end of experiment, all rats were fasted for 12h before anesthesia by intraperitoneal injection of 5% chloral hydrate (0.1 mL/10 g). Blood samples were drawn from the abdominal aorta, and the serum was obtained by centrifugation at 537 g for 15 min at 4 °C, and frozen at −80 °C until biochemical and metabolomics analysis. All the experimental protocols were approved by the animal committee of China Pharmaceutical University.

### 4.3. Biochemistry Assays

The levels of TC, TG, LDL-c, HDL-c were determined by a RT-6000 microplate reader (Shenzhen Redwood Life Technology Co. Ltd., Shenzhen, China). GraphPad Prism software version 6.01 (GraphPad Inc., La Jolla, CA, USA) was used for data analysis. Statistical analysis was carried out using two-tailed unpaired *t*-test and one-way analysis of variance (ANOVA). The criterion used for statistical significance was *p* < 0.05, *p* < 0.01 and *p* < 0.001.

### 4.4. Metabolomics Analysis Experiment

#### 4.4.1. Sample Preparation for UPLC-Q/TOF-MS

After the eighteen serum samples from the rats housed in metabolic cages, were completely thawed at 4 °C, and 100 μL of each was taken in a centrifuge tube and 300 μL of acetonitrile was added to mix. After centrifugation at 6580 g for 10 min at 4 °C, 300 μL of the supernatant was dried under gentle nitrogen stream. The residue was dissolved with 100 μL of 20% acetonitrile, which contained 10 μg/mL of chlorophenylalanine and 10 μg/mL of ketoprofen [57]. Then the mixture was centrifuged at 6580 g for 10 min at 4 °C, 80 μL of the supernatant was collected for UPLC-Q/TOF-MS. The quality control (QC) sample of serum was obtained by merging a 50 µL aliquot of each sample.

For each urine sample (100 μL), 100 μL of distilled water and 200 μL of methanol, which contained 20 μg/mL of chlorophenylalanine and 20 μg/mL of ketoprofen, were added. After centrifugation at 6580 g for 10 min at 4 °C, 350 μL of the supernatant was filtered through a 0.22 μm Millipore filter (Tianjing, China) for UPLC-Q/TOF-MS. The quality control (QC) sample of urine was obtained by merging a 50 µL aliquot of each sample.

For each feces sample (50 mg), 500 μL of 50% methanol containing 10 μg/mL of chlorophenylalanine and 10 μg/mL of ketoprofen was added and the mixture homogenized. After centrifugation at 6580 g for 10 min at 4 °C, 400 μL of the supernatant was filtered through a 0.22 μm Millipore filter for UPLC-Q/TOF-MS. The quality control (QC) sample of feces was obtained by merging a 50 mg aliquot of each sample.

#### 4.4.2. UPLC-Q/TOF-MS Analysis

Metabolomics analysis of processed serum, urine and feces samples were performed on an ACQUITY UPLC HSS T3 column (2.1 × 100 mm, 1.8 µm) at 50 °C using an Agilent 1290 UPLC-6538 Q/TOF-MS (Agilent Technologies, Santa Clara, CA, USA) in both positive and negative ion modes. The mobile phase of the positive ion mode was a mixture of 0.1% formic acid (A) and acetonitrile (B), and of the negative ion mode was a mixture of 5 mmol/L ammonium acetate (A) and acetonitrile (B). The flow rate was 0.45 mL/min. For serum, the proportion of mobile phase B was optimized as follows: 0~1 min, B (1%); 1~4 min, B (1→15%); 4~7 min, B (15→70%); 7~10 min, B (70→85%); 10~14 min, B (85→95%); 14~16 min, B (95%). The injection volume was 5 μL. For urine, the proportion was optimized as follows: 0~1 min, B (1%); 1~4 min, B (1→15%); 4~7 min, B (15→70%); 7~10 min, B (70→85%); 10~11 min, B (85→95%);11~13 min, B (95%). The injection volume was 2 μL. For feces, the proportion was optimized as follows: 0~1 min, B (1%); 1~4 min, B (1→30%); 4~6 min, B (30→70%); 6~10 min, B (70→85%); 10~12 min, B (85→95%); 12~14 min, B (95%). The injection volume was 1 μL.

Before each injection, the column was equilibrated for 4 min with 1% of phase B. Each of the QC sample was initially performed to equilibrate the column before each of the three kinds of samples and injected after every six sample injections.

MS conditions: Data was collected from *m/z* 60 to *m/z* 1000 for the positive ion mode and from *m/z* 60 to *m/z* 1100 for the negative ion mode. The capillary voltage was set at 4000 V, nebulizer pressure was set at 40 psi, the sheath gas temperature was set at 350 °C, the drying gas was set at 8 L/min at a temperature of 350 °C and the fragmentation voltage was set at 100 V.

#### 4.4.3. Metabolomics Data Analysis

The raw data obtained from the UPLC-Q/TOF-MS was converted to mzData format by the Mass Hunter Workstation Software (Version B.03.00, Agilent). Data pretreatment including peak finding, non-linear retention time alignment, filtering, alignment, matching, and identification were carried out using package XCMS in R-3.3.3. All the pretreated data was normalized by MetaboAnalyst before multivariate analysis. The normalization process was categorized into three steps, including sample normalization, data transformation and data scaling. These normalized data was then imported into SIMCA 13.0 (Umetrics, Malmö, Sweden) for multivariate analysis. The *t* test with false discovery rate correction was used to measure the significance of each metabolite. Partial least-squared discriminant analysis (PLS-DA) and orthogonal partial least-squared discriminant analysis (OPLS-DA) were conducted to identify the metabolite discrimination between the three group samples. Differential metabolites were defined with variable importance in the projection (VIP) > 1.0 obtained from OPLS-DA and p-values less than 0.05 obtained from *t* test. The trend was evaluated by fold-change values (FC), when the FC > 1, the trend was up, otherwise, the FC < 1, the trend was down. Differential metabolites were tentatively identified by database matching, i.e., Human Metabolome Database, MassHunter METLIN Metabolite PCDL (Agilent Technologies) and METLIN (http://metlin.scripps.edu). Heatmaps of differential metabolites between the ASD treated group and model group were obtained based on spearman correlation and cluster analyses. Genomes (KEGG) database and MetaboAnalyst were used to expound their related metabolic pathways.

### 4.5. Intestinal Microbiota Analysis Experiment

#### 4.5.1. DNA Extraction and High-Throughput Sequencing

Microbial DNA was extracted from the fecal samples using the E.Z.N.A.^®^ soil DNA Kit (Omega Bio-tek, Norcross, GA, USA.) according to manufacturer’s protocols. The V3-V4 hypervariable regions of the bacterial 16S rRNA were amplified with primers 338F (5′-ACTCCTACGGGAGGC AGCAG-3′) and 806R (5′-GGACTACHVGGGTWTCTAAT-3′) by a GeneAmp 9700 thermocycler (ABI, Waltham, MA, USA), following the method previously described [58]. The meta-genomic sequencing was performed on an Illumina MiSeq platform (Illumina) according to the standard protocols by Major Bio-Pharm Technology Co. Ltd. (Shanghai, China).

#### 4.5.2. Sequencing Data Analysis

The raw data obtained from meta-genomic sequencing was exported to fastq files. Data pretreatment process was quality-filtered by Trimmomatic and merged by FLASH. Operational taxonomic units (OTUs) were clustered with 97% similarity cutoff using UPARSE (version 7.1 http://drive5.com/uparse/). The taxonomy of each 16S rRNA gene sequence was analyzed by RDP Classifier algorithm (http://rdp.cme.msu.edu/) against the Silva (SSU123) 16S rRNA database using confidence threshold of 70% [59]. Alpha-diversity analysis and rarefaction curve analysis were performed using Mothur b.1.30.1. A heatmap based on the relative abundance of genus was generated using R packages 2.15. Principal coordinates analysis (PCoA) was performed using the “vegan” package in R packages 2.15. Microbial differences analysis and correlation analysis were performed using I-sanger (Majorbio Bio-Pharm Technology Co. Ltd. Shanghai, China, http://www.i-sanger.com).

## 5. Conclusions

In present study, a HFD-induced hyperlipidemia rat model was successfully established and the anti-hyperlipidemia effects of ASD were determined. Then, we employed UPLC-Q/TOF-MS and 16S rRNA gene sequencing to investigate the metabolic changes in different types of sample and the intestinal microbiome of a hyperlipidemia rat model treated with ASD. The results inferred that ASD could partially recover both metabolism dysfunction and intestinal environment through several metabolic pathways and modulation of the microbial community.

## Figures and Tables

**Table 1:** Differential metabolites in serum samples in different groups.

No.	RT (min)	Ion Mode	Formula	Mass	ppm	Identified	HMDB	MOD vs. CON			ASD vs. MOD		
								VIP	*p*-Value	Trend	VIP	*p*-Value	Trend
1	1.06	+	C_5_H_11_NO_2_S	150.0583	0.42	L-Methionine ^(a)^	HMDB00696	0.5472	0.9574	Up	1.4923	0.0297	Up
2	1.29	+	C_6_H_6_N_2_O	123.0552	0.48	Niacinamide ^(a)^	HMDB01406	0.3355	0.6946	Down	2.2065	0.0086	Up
3	1.37	−	C_6_H_13_NO_2_	130.0872	0.94	L-Leucine ^(a)^	HMDB00687	0.0028	0.4151	Down	1.3135	0.0203	Up
4	1.50	+	C_8_H_9_NO	136.0757	−0.11	2-Phenylacetamide ^(a)^	HMDB10715	0.6854	0.8185	Up	1.6481	0.0191	Up
5	2.37	+	C_10_H_19_NO_4_	218.1388	−2.01	Propionyl-L-carnitine	HMDB00824	1.5639	0.0111	Up	1.0147	0.2684	Down
6	4.90	−	C_11_H_11_NO_2_	188.0714	2.47	3-Indolepropionic acid	HMDB02302	1.1230	0.0195	Down	1.2291	0.0791	Up
7	6.75	−	C_24_H_40_O_5_	407.2809	−1.40	Cholic acid	HMDB00619	1.6343	0.0001	Down	0.2483	0.8277	Up
8	7.12	+	C_21_H_30_O_4_	347.2222	−2.02	Corticosterone ^(a)^	HMDB01547	0.7386	0.0776	Down	1.3824	0.0340	Up
9	7.56	+	C_21_H_39_NO_4_	370.2957	−0.07	*cis*-5-Tetradecenoylcarnitine	HMDB02014	1.6167	0.0064	Up	0.6820	0.3477	Down
10	7.91	+	C_18_H_38_NO_5_P	380.2567	0.04	Sphingosine 1-phosphate	HMDB00277	1.6649	0.0088	Up	0.3627	0.4348	Down
11	7.98	+	C_22_H_46_NO_7_P	468.3092	0.49	LysoPC(14:0)	HMDB10379	1.5328	0.0008	Down	0.1945	0.9340	Up
12	8.04	+	C_18_H_40_NO_5_P	382.2722	0.66	Sphinganine 1-phosphate	HMDB01383	1.8018	0.0005	Up	0.3383	0.8381	Down
13	8.09	+	C_26_H_48_NO_7_P	518.3246	0.04	LysoPC(18:3(9Z,12Z,15Z))	HMDB10388	1.5738	0.0005	Down	0.6690	0.6562	Down
14	8.17	+	C_26_H_46_NO_7_P	516.3065	−4.89	LysoPC(18:4(6Z,9Z,12Z,15Z))	HMDB10389	1.7202	0.0002	Down	0.1072	0.7307	Up
15	8.19	+	C_25_H_47_NO_4_	426.3584	0.24	Elaidic carnitine	HMDB06464	1.7357	0.0007	Up	0.0138	0.8087	Up
16	8.27	+	C_23_H_48_NO_7_P	482.3249	0.66	LysoPC(15:0)	HMDB10381	1.6095	0.0013	Down	0.1400	0.9923	Down
17	8.33	+	C_30_H_50_NO_7_P	568.3405	0.49	LysoPC(22:6(4Z,7Z,10Z,13Z,16Z,19Z))	HMDB10404	1.6564	0.0012	Down	0.5903	0.9626	Down
18	8.50	+	C_25_H_50_NO_7_P	508.3403	1.17	LysoPE(20:1(11Z)/0:0)	HMDB11512	1.6807	0.0001	Down	0.6203	0.6862	Down
19	8.52	+	C_30_H_52_NO_7_P	570.3553	0.11	LysoPC(22:5(7Z,10Z,13Z,16Z,19Z))	HMDB10403	1.7786	0.0004	Down	0.7303	0.3004	Up
20	8.53	+	C_25_H_49_NO_4_	428.3740	0.08	DL-Stearoylcarnitine	HMDB00848	1.7399	0.0029	Up	1.3606	0.2079	Down
21	8.56	−	C_21_H_44_NO_7_P	452.2790	−1.67	LysoPE(16:0) ^(a)^	HMDB11473	0.9519	0.0071	Down	1.4901	0.0103	Down
22	8.98	+	C_30_H_54_NO_7_P	572.3715	−1.10	LysoPC(22:4(7Z,10Z,13Z,16Z))	HMDB10401	1.3823	0.0251	Down	0.2440	0.8110	Up
23	9.08	+	C_25_H_52_NO_7_P	510.3565	0.66	LysoPC(17:0)	HMDB12108	1.3741	0.0251	Down	1.2498	0.3201	Down
24	9.16	−	C_20_H_30_O_2_	301.2175	−0.72	Eicosapentaenoic Acid	HMDB01999	1.4389	0.0096	Down	1.3958	0.1345	Down
25	9.43	+	C_23_H_48_NO_7_P	482.3249	−0.78	LysoPE(18:0) ^(a)^	HMDB11130	1.1933	0.3201	Up	1.8859	0.0109	Down
26	9.64	−	C_27_H_46_O_4_S	465.3058	−2.98	Cholesterol sulfate	HMDB00653	1.2191	0.0232	Down	1.1410	0.1093	Up
27	10.50	−	C_20_H_34_O_2_	305.2489	−0.90	Dihomo-γ-linolenic Acid	HMDB02925	1.6323	0.0092	Up	0.6575	0.6970	Down
28	11.39	−	C_20_H_36_O_2_	307.2644	−0.61	Eicosadienoic Acid	HMDB05060	1.4032	0.0333	Up	0.9846	0.3784	Down
29	11.84	+	C_18_H_34_O_2_	283.2636	−0.13	Oleic Acid	HMDB00207	1.4714	0.0450	Up	1.0214	0.3627	Down

RT: Retention time; HMDB: Human Metabolome Database; VIP: variable importance in the projection was obtained from orthogonal partial least square-discriminant analysis (OPLS-DA) model; *p*-value: values were calculated from *t*-test; CON: the chow-diet group; MOD: the model group; ASD: the ASD treated group; ^(a)^: differential metabolites between the ASD treated group and MOD group based on the VIP > 1 and *p*-value < 0.05.

**Table 2:** Differential metabolites in urine samples in different groups.

No.	RT (min)	Ion Mode	Formula	Mass	ppm	Identified	HMDB	MOD vs. CON			ASD vs. MOD		
								VIP	*p*-Value	Trend	VIP	*p*-Value	Trend
1	0.71	−	C_5_H_6_O_5_	145.0141	2.41	Oxoglutaric acid ^(a)^	HMDB00208	0.1924	0.7928	Down	1.2680	0.0053	Down
2	0.81	+	C_7_H_8_N_2_O	137.0708	−4.22	2-Methylnicotinamide ^(a)^	HMDB03152	1.7566	0.0143	Down	1.5038	0.0129	Up
3	1.22	+	C_4_H_5_N_3_O	112.0507	−3.01	Cytosine ^(a)^	HMDB00630	0.9619	0.0576	Up	1.1377	0.0352	Down
4	1.46	+	C_5_H_7_N_3_O	126.0662	−4.26	5-Methylcytosine ^(a)^	HMDB02894	0.8145	0.0602	Up	1.1879	0.0127	Down
5	1.73	−	C_7_H_6_O_2_	121.0294	3.76	Benzoic acid	HMDB01870	1.4449	0.0056	Down	1.2835	0.0889	Up
6	1.87	+	C_6_H_13_NO_2_	132.1019	−4.09	L-Leucine ^(a)^	HMDB00687	1.0040	0.0171	Up	1.3214	0.0136	Down
7	2.07	+	C_5_H_6_N_2_O_2_	127.0501	−4.94	Thymine ^(a)^	HMDB00262	0.8082	0.5633	Down	1.3919	0.0166	Up
8	3.08	+	C_9_H_11_NO_2_	166.0862	−3.78	L-Phenylalanine ^(a)^	HMDB00159	1.1454	0.0018	Up	1.2179	0.0118	Down
9	3.43	−	C_8_H_15_NO_3_	172.0977	1.89	Acetyl-L-leucine ^(a)^	HMDB11756	1.0070	0.0622	Up	1.1189	0.0048	Down
10	3.55	−	C_10_H_11_NO_3_	385.1409	2.98	Methylhippuric acid ^(a)^	HMDB11723	0.5680	0.3307	Up	1.4407	0.0327	Up
11	3.66	−	C_13_H_16_O_7_	283.0825	2.48	*p*-Cresol glucuronide	HMDB11686	1.8653	0.0287	Up	0.7992	0.0716	Down
12	3.72	+	C_12_H_17_N_5_O_5_	312.1306	−0.30	N2,N2-Dimethylguanosine ^(a)^	HMDB04824	0.8670	0.0553	Up	1.0178	0.0155	Down
13	4.00	+	C_11_H_12_N_2_O_2_	205.0972	−2.48	L-Tryptophan ^(a)^	HMDB00929	0.9814	0.0171	Up	1.3191	0.0090	Down
14	4.13	−	C_9_H_11_NO_7_S	276.0186	2.92	DOPA sulfate ^(a)^	HMDB02028	0.9182	0.0056	Up	1.3098	0.0033	Down
15	4.77	+	C_8_H_5_NO_2_	148.0392	−4.26	Indole-5,6-quinone ^(a)^	HMDB06779	1.0051	0.0183	Up	1.2833	0.0209	Down
16	5.44	+	C_9_H_7_NO_2_	162.0549	−3.80	2-Indolecarboxylic acid	HMDB02285	1.8083	0.0195	Down	0.1418	0.6702	Down
17	5.64	+	C_8_H_17_NO_3_	174.1124	−3.31	Hexanoylglycine ^(a)^	HMDB00701	0.9603	0.1345	Up	1.1101	0.0092	Down
18	6.26	+	C_10_H_9_NO_2_	176.0706	−3.24	3-Indoleacetic Acid ^(a)^	HMDB00197	0.6993	0.3707	Up	1.0799	0.0194	Down
19	10.59	−	C_16_H_32_O_2_	255.2330	2.13	Palmitic acid ^(a)^	HMDB00220	1.4322	0.3588	Down	1.5776	0.0266	Up

RT: Retention time; HMDB: Human Metabolome Database; VIP: variable importance in the projection was obtained from orthogonal partial least square-discriminant analysis (OPLS-DA) model; *p*-value: values were calculated from *t*-test; CON: the chow-diet group; MOD: the model group; ASD: the ASD treated group; ^(a)^: differential metabolites between the ASD treated group and MOD group based on the VIP > 1 and *p*-value < 0.05.

**Table 3:** Differential metabolites in feces samples in different groups.

No.	RT (min)	Ion Mode	Formula	Mass	ppm	Identified	HMDB	MON vs. CON			ASD vs. MOD		
								VIP	*p*-Value	Trend	VIP	*p*-Value	Trend
1	0.73	−	C_5_H_9_NO_4_	146.0457	1.33	L-Glutamate ^(a)^	HMDB00148	0.0031	0.4466	Up	1.8155	0.0119	Down
2	0.88	−	C_6_H_8_N_2_O_2_	139.0512	0.87	1,4-Methylimidazoleacetic acid	HMDB02820	1.0421	0.0096	Up	0.9254	0.1370	Down
3	3.21	+	C_8_H_11_N	122.0965	−0.34	1-Phenylethylamine ^(a)^	HMDB02017	0.7189	0.0992	Up	1.5301	0.0095	Up
4	3.64	+	C_10_H_12_N_2_	161.1072	0.83	Tryptamine	HMDB00303	1.3791	0.0087	Up	0.6520	0.4670	Down
5	5.97	−	C_24_H_40_O_4_	391.2889	−8.03	Deoxycholic Acid ^(a)^	HMDB00518	1.3663	0.0007	Up	1.2736	0.0309	UP
6	8.65	−	C_16_H_30_O_2_	253.2174	−0.55	*cis*-9-palmitoleic acid ^(a)^	HMDB03229	0.7390	0.2962	Up	1.6506	0.0419	Down

RT: Retention time; HMDB: Human Metabolome Database; VIP: variable importance in the projection was obtained from orthogonal partial least square-discriminant analysis (OPLS-DA) model; *p*-value: values were calculated from *t*-test; CON: the chow-diet group; MOD: the model group; ASD: the ASD treated group; ^(a)^: differential metabolites between the ASD treated group and MOD group based on the VIP > 1 and *p*-value < 0.05.

## Supplementary Materials

The following are available online at https://www.mdpi.com/1420-3049/24/7/1268/s1, File 1: Figure S1: Representative total ion chromatograms (TIC) of serum samples in positive ion mode (A) and negative ion mode (B) with identified differential metabolites. Figure S2: Metabolic pathway analysis of differential metabolites of serum samples between the ASD treated group and the model group (a: valine, leucine and isoleucine biosynthesis; b: nicotinate and nicotinamide metabolism; c: cysteine and methionine metabolism; d: steroid hormone biosynthesis; e: aminoacyl-tRNA biosynthesis; f: phenylalanine metabolism; g: valine, leucine and isoleucine degradation). Figure S3: Representative total ion chromatograms (TIC) of urine samples in positive ion mode (A) and negative ion mode (B) with identified differential metabolites. Figure S4: Metabolic pathway analysis of differential metabolites of urine samples between the ASD treated group and the model group (a: phenylalanine, tyrosine and tryptophan biosynthesis; b: phenylalanine metabolism; c: valine, leucine and isoleucine biosynthesis; d: tryptophan metabolism; e: citrate cycle [TCA cycle]; f: alanine, aspartate and glutamate metabolism; g: pyrimidine metabolism; h: aminoacyl-tRNA biosynthesis; i: d-glutamine and d-glutamate metabolism; j: butanoate metabolism; k: fatty acid elongation in mitochondria; l: valine, leucine and isoleucine degradation; m: Fatty acid metabolism). Figure S5: Representative total ion chromatograms (TIC) of feces samples in positive ion mode (A) and negative ion mode (B) with identified differential metabolites. Figure S6: Metabolic pathway analysis of differential metabolites of feces samples between the ASD treated group and the model group (a: d-glutamine and d-glutamate metabolism; b: alanine, aspartate and glutamate metabolism; c: arginine and proline metabolism; d: glutathione metabolism; e: nitrogen metabolism; f: histidine metabolism; g: butanoate metabolism; h: porphyrin and chlorophyll metabolism; i: primary bile acid biosynthesis; j: aminoacyl-tRNA biosynthesis). Figure S7: Rarefaction curves among the three groups based on the SOBS (A) and Shannon index (B). Figure S8: The Bray-Curtis PCoA analysis of the microbiota composition at genus (A), species (B) and OUT (C) levels between the three groups. Figure S9: Community heatmap of key OTUs based on genus-level changes among the three groups. The relative abundance of each genus was indicated by a gradient of color from green (low abundance) to red (high abundance). Complete linkage clustering of samples was based on the genus composition and abundance.
