# Supplementary material for: Akebia Saponin D Regulates the Metabolome and Intestinal Microbiota in High Fat Diet-Induced Hyperlipidemic Rats
Source: Molecules. 2019 Apr 1;24(7):1268. doi: 10.3390/molecules24071268 (PMC6479315; doi:10.3390/molecules24071268)
Supplement: Supplementary file 1 [file molecules-24-01268-s001.pdf]

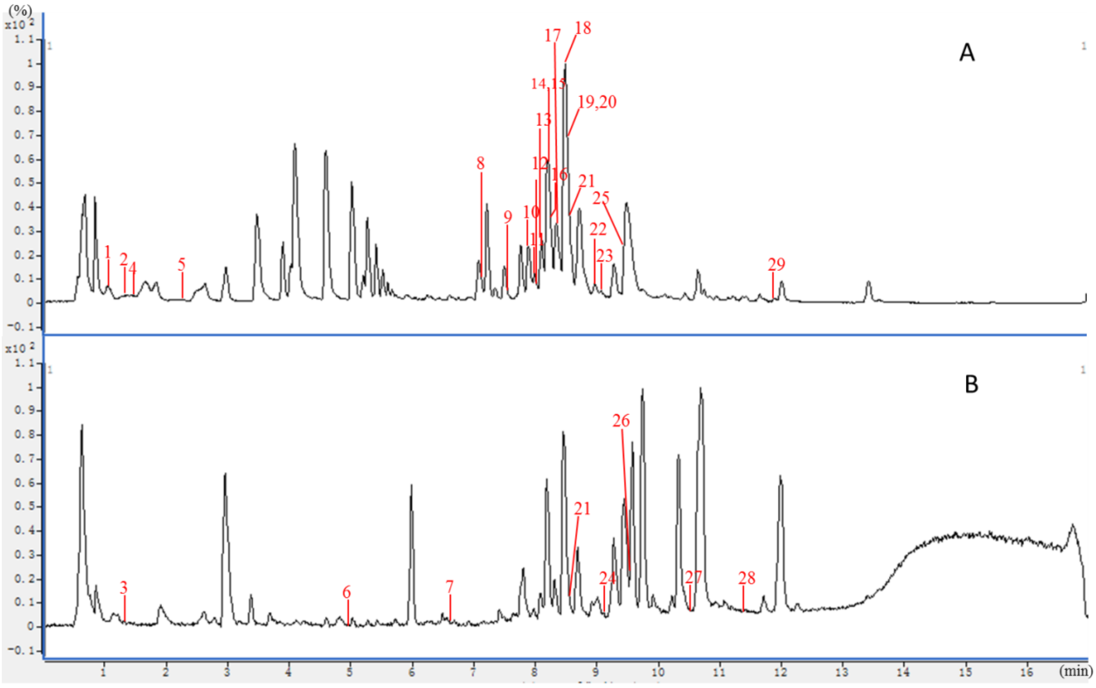

Fig.S1 Representative total ion chromatograms (TIC) of serum samples in positive ion mode (**A**) and negative ion mode (**B**) with identified differential metabolites.

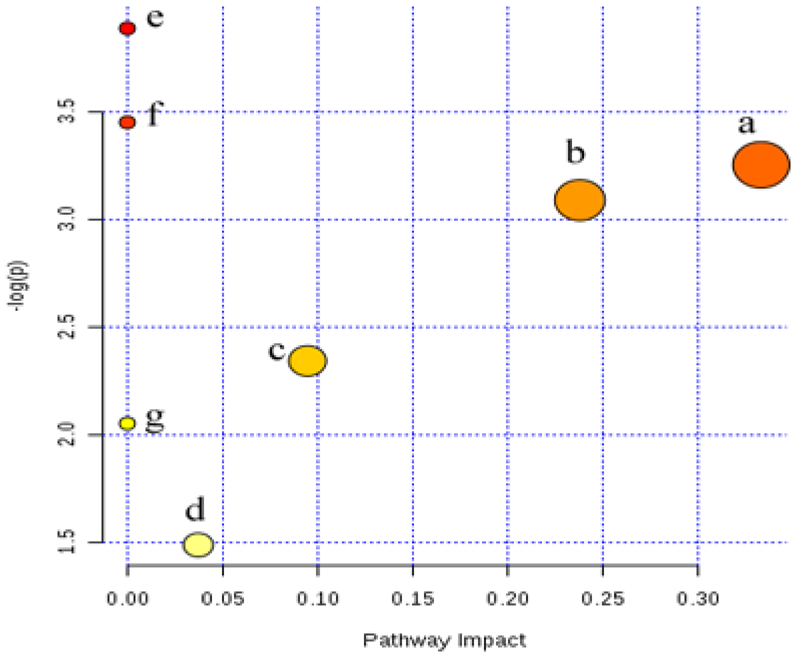

Fig.S2 Metabolic pathway analysis of differential metabolites of serum samples between the ASD treated group and the model group (a: Valine, leucine and isoleucine biosynthesis; b: Nicotinate and nicotinamide metabolism; c: Cysteine and methionine metabolism; d: Steroid hormone biosynthesis; e: Aminoacyl-tRNA biosynthesis; f: Phenylalanine metabolism; g: Valine, leucine and isoleucine degradation).

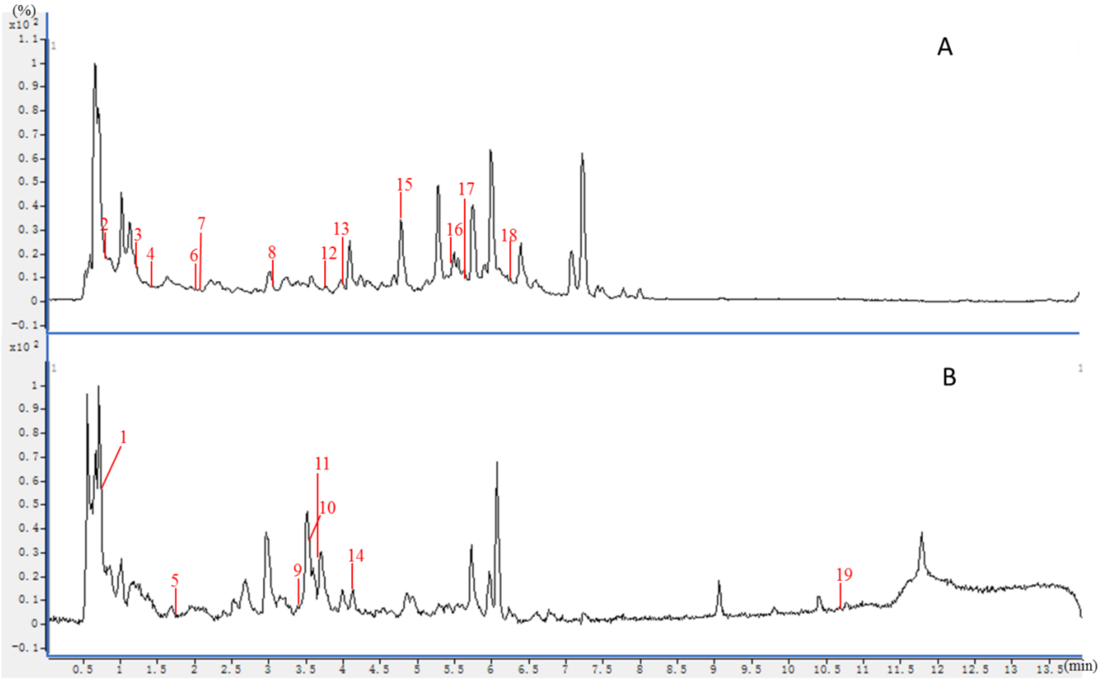

Fig.S3 Representative total ion chromatograms (TIC) of urine samples in positive ion mode (**A**) and negative ion mode (**B**) with identified differential metabolites.

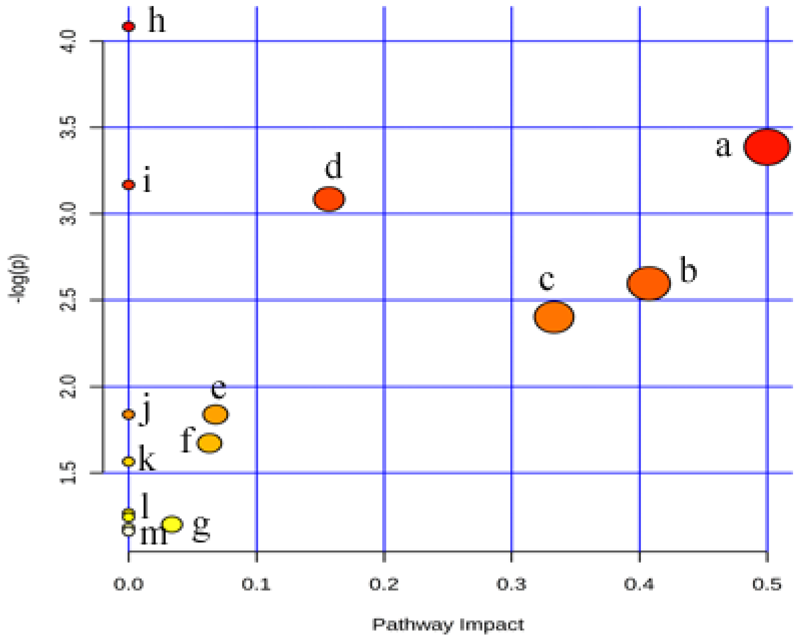

Fig.S4 Metabolic pathway analysis of differential metabolites of urine samples between the ASD treated group and the model group (a: Phenylalanine, tyrosine and tryptophan biosynthesis; b: Phenylalanine metabolism; c: Valine, leucine and isoleucine biosynthesis; d: Tryptophan metabolism; e: Citrate cycle [TCA cycle]; f: Alanine, aspartate and glutamate metabolism; g: Pyrimidine metabolism; h: Aminoacyl-tRNA biosynthesis; i: D-Glutamine and D-glutamate metabolism; j: Butanoate metabolism; k: Fatty acid elongation in mitochondria; l: Valine, leucine and isoleucine degradation; m: Fatty acid metabolism).

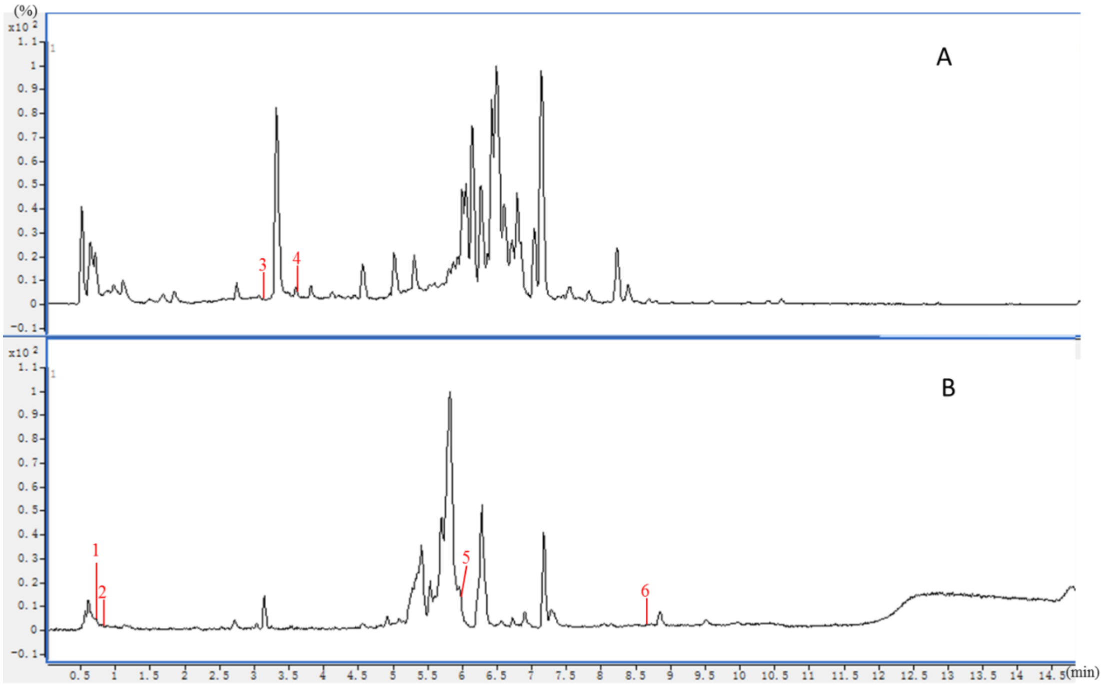

Fig.S5 Representative total ion chromatograms (TIC) of feces samples in positive ion mode (**A**) and negative ion mode (**B**) with identified differential metabolites.

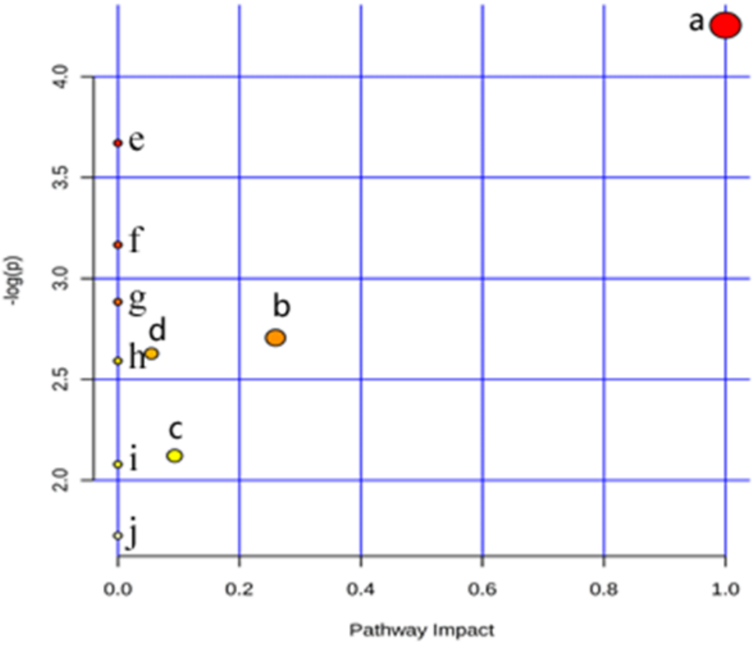

Fig.S6 Metabolic pathway analysis of differential metabolites of feces samples between the ASD treated group and the model group (a: D-Glutamine and D-glutamate metabolism; b: Alanine, aspartate and glutamate metabolism; c: Arginine and proline metabolism; d: Glutathione metabolism; e: Nitrogen metabolism; f: Histidine metabolism; g: Butanoate metabolism; h: Porphyrin and chlorophyll metabolism; i: Primary bile acid biosynthesis; j: Aminoacyl-tRNA biosynthesis).

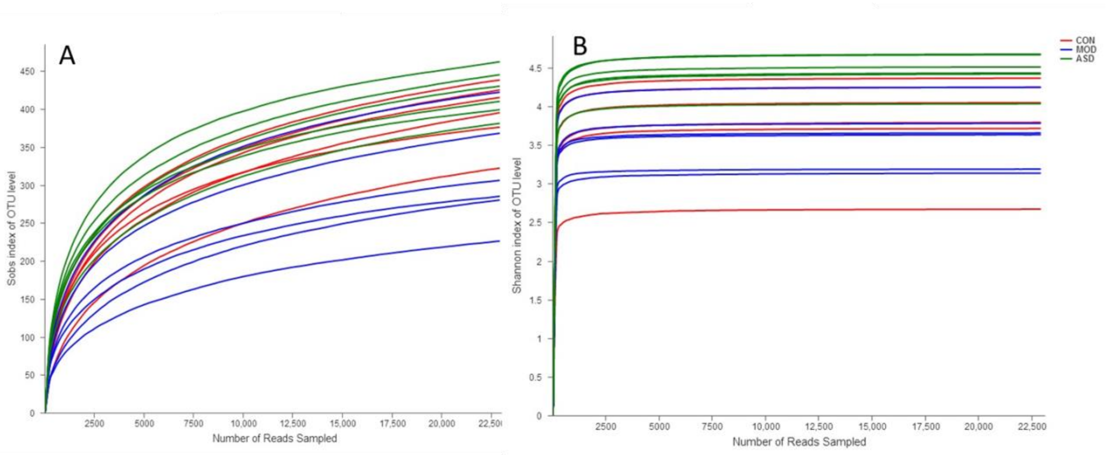

Fig.S7 Rarefaction curves among the three groups based on the sobs(**A**) and Shannon index(**B**) (CON = the chow diet group, n=6; MOD = high-fat diet model group, n=6; ASD = ASD treated group, n=6)

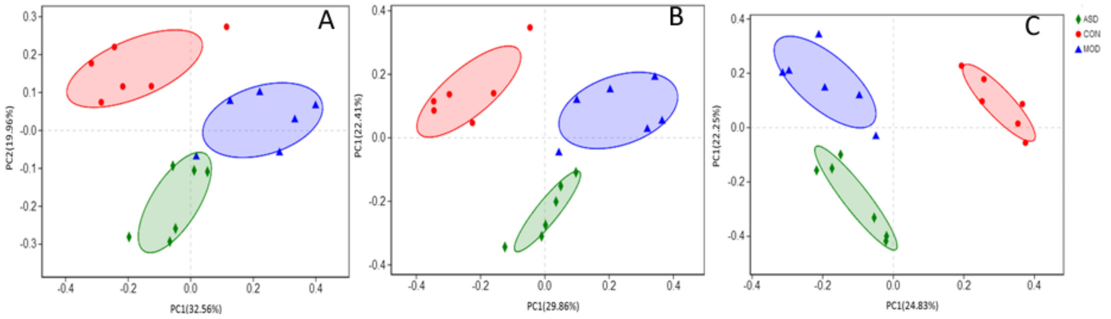

Fig.S8 The bray-curtis PCoA analysis of the microbiota composition at genus (**A**), species (**B**) and OUT (**C**) levels between the three groups. (CON = the chow diet group, n=6; MOD = high-fat diet model group, n=6; ASD = ASD treated group, n=6)

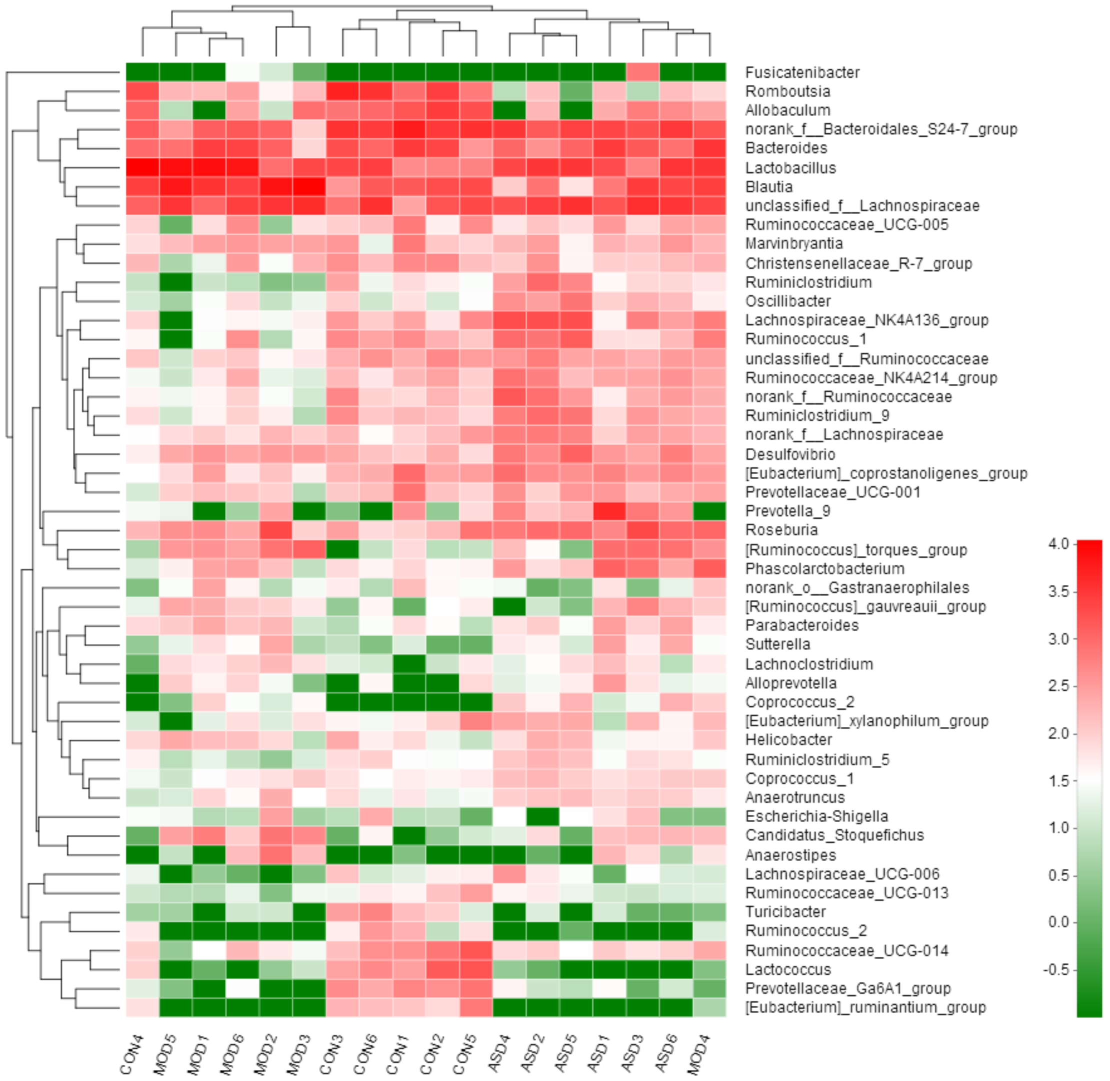

Fig.S9 Community heatmap of key OTUs based on genus-level changes among the three groups. The relative abundance of each genus was indicated by a gradient of color from green (low abundance) to red (high abundance). Complete linkage clustering of samples was based on the genus composition and abundance. (CON = the chow diet group, n=6; MOD = high-fat diet model group, n=6; ASD = ASD treatment group, n=6)
